# Difference of Precocious Puberty Between Before and During the COVID-19 Pandemic: A Cross-Sectional Study Among Shanghai School-Aged Girls

**DOI:** 10.3389/fendo.2022.839895

**Published:** 2022-03-21

**Authors:** Yao Chen, Jianyong Chen, Yijun Tang, Qianwen Zhang, Yirou Wang, Qun Li, Xin Li, Zihan Weng, Ju Huang, Xiumin Wang, Shijian Liu

**Affiliations:** ^1^ Department of Endocrinology, Genetics and Metabolism, Shanghai Children’s Medical Center, School of Medicine, Shanghai Jiao Tong University, Shanghai, China; ^2^ Department of Otorhinolaryngology-Head and Neck Surgery, Xinhua Hospital, School of Medicine, Shanghai Jiao Tong University, Shanghai, China; ^3^ Department of Information Technology, Shanghai Children’s Medical Center, School of Medicine, Shanghai Jiao Tong University, Shanghai, China; ^4^ Center for Brain Science, Shanghai Children’s Medical Center, School of Medicine, Shanghai Jiao Tong University, Shanghai, China; ^5^ Department of Clinical Epidemiology and Biostatistics, Children Health Advocacy Institute, Shanghai Children’s Medical Center, School of Medicine, Shanghai Jiao Tong University, Shanghai, China; ^6^ School of Public Health, School of Medicine, Shanghai Jiao Tong University, Shanghai, China

**Keywords:** precocious puberty, girl, COVID-19, ghrelin, MKRN3

## Abstract

**Objective:**

To compared the incidence rates and clinical features of precocious girls before and during the COVID-19 pandemic among Shanghai school-aged girls, and explored the potential mechanisms.

**Methods:**

This cross-sectional study collected medical data about precocious girls between 2016 and 2020 from Shanghai Children’s Medical Center. Data of inpatient precocious girls from March to August in 2016-2019 (n=246) and 2020 (n=237) were collected. Subjects with abnormal brain and pituitary gland MRI reports, other endocrine diseases or chronic diseases were excluded. Finally, 209 precocious girls were included in the 2016-2019 group and 191 precocious girls were include in the 2020 group. Monthly incidence rates and clinical features were compared between before and during the COVID-19 pandemic. Linear regression models were used to examine the associations between biomarkers to explore the potential mechanisms.

**Results:**

Monthly incidence rates of precocious puberty in outpatient girls from March to December 2020 (0.44-1.36%) and in inpatient girls from March to August 2020 (27.04-47.83%) were higher than those in 2016-2019 (0.30-0.52% and 10.53-18.42%, respectively). Serum concentrations of GnRH were higher in the 2020 group than in the 2016-2019 group (2.81 vs 1.99 mg/L). Serum concentrations of MKRN3 (1.02 vs 1.93 ng/ml) and ghrelin (0.38 vs 0.88 ng/ml) were lower in the 2020 group than in the 2016-2019 group. Moreover, the serum concentration of ghrelin was positively associated with the serum concentration of MKRN3 [*β=*0.891 (*95% CI*, 0.612, 1.171); *p<*0.001].

**Conclusions:**

These findings suggest an increased incidence of precocious puberty during the COVID-19 pandemic among Shanghai school-aged girls, which may be associated with decreased serum concentrations of MKRN3 and ghrelin, and indicated ghrelin as a potential regulatory mechanism of puberty.

## Introduction

Since early December 2019, coronavirus 2019 (COVID-19) has spread rapidly and widely worldwide (1, 2). It was suggested that children are less susceptible to COVID-19 than adults (3), and pediatric patients have less severe clinical manifestations than adult patients (2). However, increasing evidence has demonstrated that control policies for COVID-19, such as enforced social distancing, school closures, online courses and lifestyle changes that reduce physical activities, may lead to other serious problems in children (4).

Among these problems, the increased incidence of precocious puberty in girls attracted our attention (3, 5). Li et al. found that the spectrum of disease for children changed dramatically before and after the breakout of COVID-19 in Hangzhou (3). It showed that the greatest increases in visits were for problems related to precocious and accelerated puberty (3). Stagi et al. also suggested that there was an increased incidence of precocious and accelerated puberty in girls during and after the Italian lockdown for the COVID-19 pandemic (5). Compared with previous years, precocious girls during and after lockdown showed a faster rate of pubertal progression (5). Shanghai is an international city with a large domestic and overseas population. The task of epidemic prevention is still grim, and policies for pandemic control remain strict in Shanghai. However, very few studies have investigated the changes in precocious incidence and clinical features before and during COVID-19 in school-aged girls in Shanghai.

In this study, we analyzed data on outpatients and inpatients in 2016-2020 from Shanghai Children’s Medical Center (SCMC) to compare the incidence rates and clinical features of precocious girls before and during the COVID-19 pandemic among Shanghai school-aged girls. Furthermore, serum concentrations of biomarkers were detected for inpatient precocious girls to explore the potential mechanisms.

## Methods

### Study Design and Participants

This study was conducted in SCMC, Shanghai Jiao Tong University School of Medicine, Shanghai, China. SCMC is one of the National Children’s Medical Centers in China, accounting for 15-20% of the total number of pediatric patients in Shanghai, with more than 1,700,000 visits in the outpatient department and more than 35,000 visits in the inpatient ward per year.

Participants were diagnosed with precocious puberty according to the onset of breast development (Tanner stages≥B2) before the chronological age (CA) of 8 years (6). Precocious girls who showed slow progress of puberty were followed in the outpatient department. Other girls who showed accelerated puberty were admitted in the endocrinology ward for further examinations. Data of these precocious girls in 2016-2020 from SCMC were recorded.

Girls younger than 6 years were more susceptible to intracranial pathology (7). We collected data on precocious girls equal or older than 6 years from the endocrinology ward from March to August in 2016-2020. Detailed data of physical and laboratory examinations were recorded for these inpatient precocious girls, including anthropometric measurements, pubertal staging, direct radiography of the left hand and wrist, pelvic ultrasonography, magnetic resonance imaging (MRI) of the brain and pituitary gland and gonadotropin-releasing hormone (GnRH) stimulation tests.

**Figure 1:**
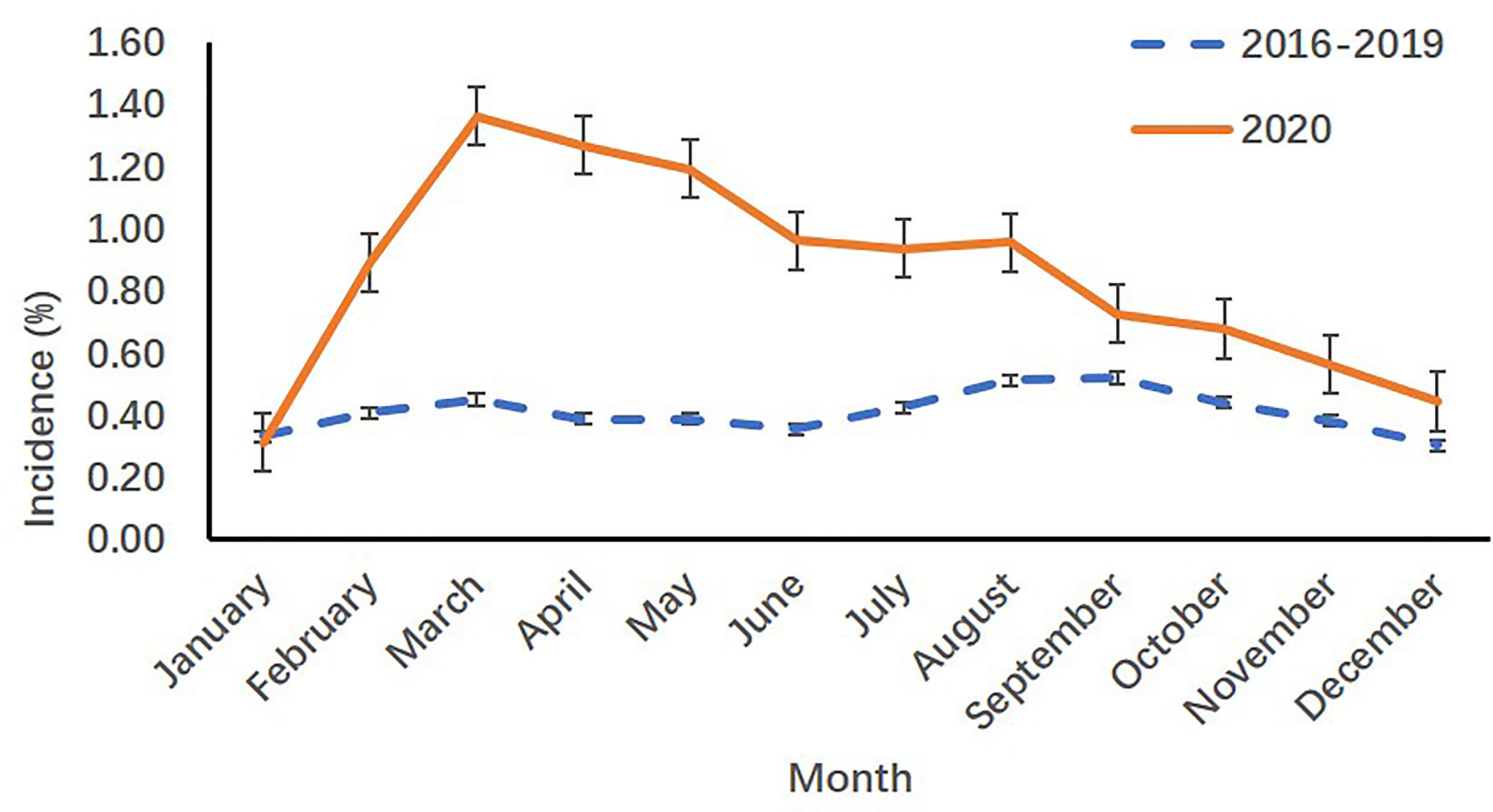
Monthly incidence rates of precocious girls before (2016-2019) and during (2020) the COVID-19 pandemic in the outpatient department.

Subjects with any one of the following conditions were excluded: 1) abnormal brain and pituitary gland MRI reports, including Rathke’s cleft cysts, pineal cysts, arachnoid cysts, hamartoma and germinoma; 2) other endocrine diseases, including hypothyroidism, hyperthyroidism, short stature, diabetes and adrenal disease; and 3) chronic diseases, including chronic nephrosis, asthma, epilepsy and hematological disease. After that, inpatient precocious girls were included in the 2016-2019 group and 2020 group (**Figure 1**), and their clinical features were compared.

### Physical examination

The body weight (kg) and height (cm) of all subjects were measured and recorded using the same type of apparatus and following the standard procedures recommended by Cameron (8). Body mass index (BMI) was calculated by the equation of weight in kilograms (kg) divided by height in meters squared (m2). Secondary sexual characteristics of these subjects were evaluated and recorded by endocrinologists from the Department of Endocrinology using Tanner stages (9).

### Laboratory examination

Bone age (BA) (10), pelvic ultrasonography (11), magnetic resonance imaging (MRI) of the brain and pituitary gland, gonadotropin-releasing hormone (GnRH)-stimulation test, serum concentrations of sex hormone-binding globulin (SHBG) and hormones [estradiol (E2), luteinizing hormone (LH), follicle-stimulating hormone (FSH), GnRH, kisspeptin, makorin ring finger protein 3 (MKRN3), leptin and ghrelin)] were detected for inpatient precocious girls in the 2016-2019 group and 2020 group (**Figure 1**).

Gonadorelin acetate (Gonadorelin, BBCA, Anhui, China) was used for the GnRH stimulation test, which was conducted between 8 am and 10 am in a fasting state for all of the cases in the 2016-2019 group and 2020 group, by intravenous injection of gonadorelin acetate at a dose of 2.5 μg/kg body weight (the maximum total dose was 100 μg) (12). Venous blood samples were collected before the injection of gonadorelin acetate to detect the serum concentrations of SHBG, E2, LH, FSH, GnRH, kisspeptin, MKRN3, leptin and ghrelin at 0, 30, and 60 min after the injection of gonadorelin acetate to detect the serum concentrations of LH and FSH at 30 and 60 min.

Serum was separated from the blood samples less than 1 hour after collection. For the concentrations of SHBG, E2, LH and FSH, serum samples were detected just after being collected. For the concentrations of GnRH, kisspeptin, MKRN3, leptin and ghrelin, serum samples were stored in the biobank at -80°C until the detection. We lost some of our samples because of updates to the biobank electronic system (**Figure 1**). Serum samples were detected by the clinical laboratory of SCMC. Serum concentrations of SHBG, E2, LH and FSH were detected by chemiluminescence immunoassay (CMIA; Abbott Diagnostics, IL, USA) on an Architect i2000SR (Abbott Diagnostics), with detection limits of 0.02 nmol/L, 10 pg/ml, 0.09 IU/L and 0.05 IU/L, respectively; intra-assay and inter-assay coefficients of variation (CV) were less than 6.4% and 8.4%, respectively. Serum concentrations of GnRH, kisspeptin, MKRN3, leptin and ghrelin were measured using enzyme-linked immunosorbent assay (ELISA) by commercial human ELISA kits (BFS, Beijing, China), with detection limits of 20 ng/L, 5 ng/L, 10 ng/L, 0.05 ng/ml and 5 ng/L, respectively; intra-assay and inter-assay CV were less than 10% and 12%, respectively.

### Questionnaire Survey

According to questionnaires used in our previous investigations (13), a structured questionnaire was designed to investigate lifestyles of precocious girls during the COVID-19 pandemic. Face-to-face interviews were conducted for inpatient precocious girls in the 2020 group through the questionnaire, including detailed information about dietary pattern, rate of weight gain, sleep duration, amount of exercise and screen exposure duration during the COVID-19 pandemic. Totally, 23.56% (45/191) of precocious girls and their parents completed the questionnaire due to the inconvenient return visit during the COVID-19 pandemic.

### Statistical Analysis

The monthly incidence of precocious puberty in outpatient girls was calculated by monthly visits of precocious girls divided by monthly visits of all patients in the outpatient department. Monthly incidence of precocious puberty in inpatient girls was calculated by monthly visits of precocious girls divided by monthly visits of all patients in the endocrinology ward. Comparisons among groups were analyzed using chi-square (*χ^2^*) tests for categorical variables and Wilcoxon rank sum tests for continuous variables due to their skewed distributions. The relationships of serum ghrelin with serum GnRH and MKRN3 concentrations in precocious girls were analyzed using linear regression models, adjusting for girls’ age, BMI and the menarche age of their mothers (14). Serum concentrations of GnRH, MKRN3 and ghrelin were transformed to log10 scale due to their skewed distributions. Statistical analyses were performed with SPSS 25.0 (SPSS Inc., Chicago, IL, USA) based on two-tailed tests, and statistical significance was set at *p*< 0.05.

### Ethics

All participating parents provided written informed consent, and all research activities were approved by the institutional review board of SCMC, Shanghai Jiao Tong University School of Medicine (approval number: SCMCIRB-K2021014-1).

## Results

When compared with the incidence in 2016-2019 years, both the monthly visits and monthly incidence rates of precocious girls increased from March to December 2020 in the outpatient department of SCMC (*p*<.001) [(**Table 1**) and (**Figure 1**)].

**Table 1:** Monthly visits and monthly incidence rates of precocious girls before (2016-2019) and during (2020) the COVID-19 pandemic in the outpatient department.

Month	Average visits of patients before the COVID-19 pandemic (2016-2019)			Visits of patients during the COVID-19 pandemic (2020)			*χ2*	*p*
	Precocious girls	All patients	Incidence (%)	Precocious girls	All patients	Incidence (%)		
January	454	136950	0.33	398	127277	0.31	0.73	0.394
February	395	97130	0.41	244	27386	0.89	98.14	**<0.001**
March	573	127022	0.45	636	46740	1.36	409.16	**<0.001**
April	496	127545	0.39	863	68025	1.27	497.60	**<0.001**
May	540	139369	0.39	1021	85561	1.19	499.53	**<0.001**
June	479	135167	0.35	917	95455	0.96	341.80	**<0.001**
July	606	143060	0.42	1056	112767	0.94	256.97	**<0.001**
August	725	141594	0.51	1135	118776	0.96	179.17	**<0.001**
September	664	127436	0.52	856	118034	0.73	41.51	**<0.001**
October	582	132369	0.44	786	115948	0.68	64.02	**<0.001**
November	559	146758	0.38	695	123445	0.56	48.13	**<0.001**
December	474	157299	0.30	663	149415	0.44	42.07	**<0.001**

Bold indicates p < 0.05.

**Figure 2:**
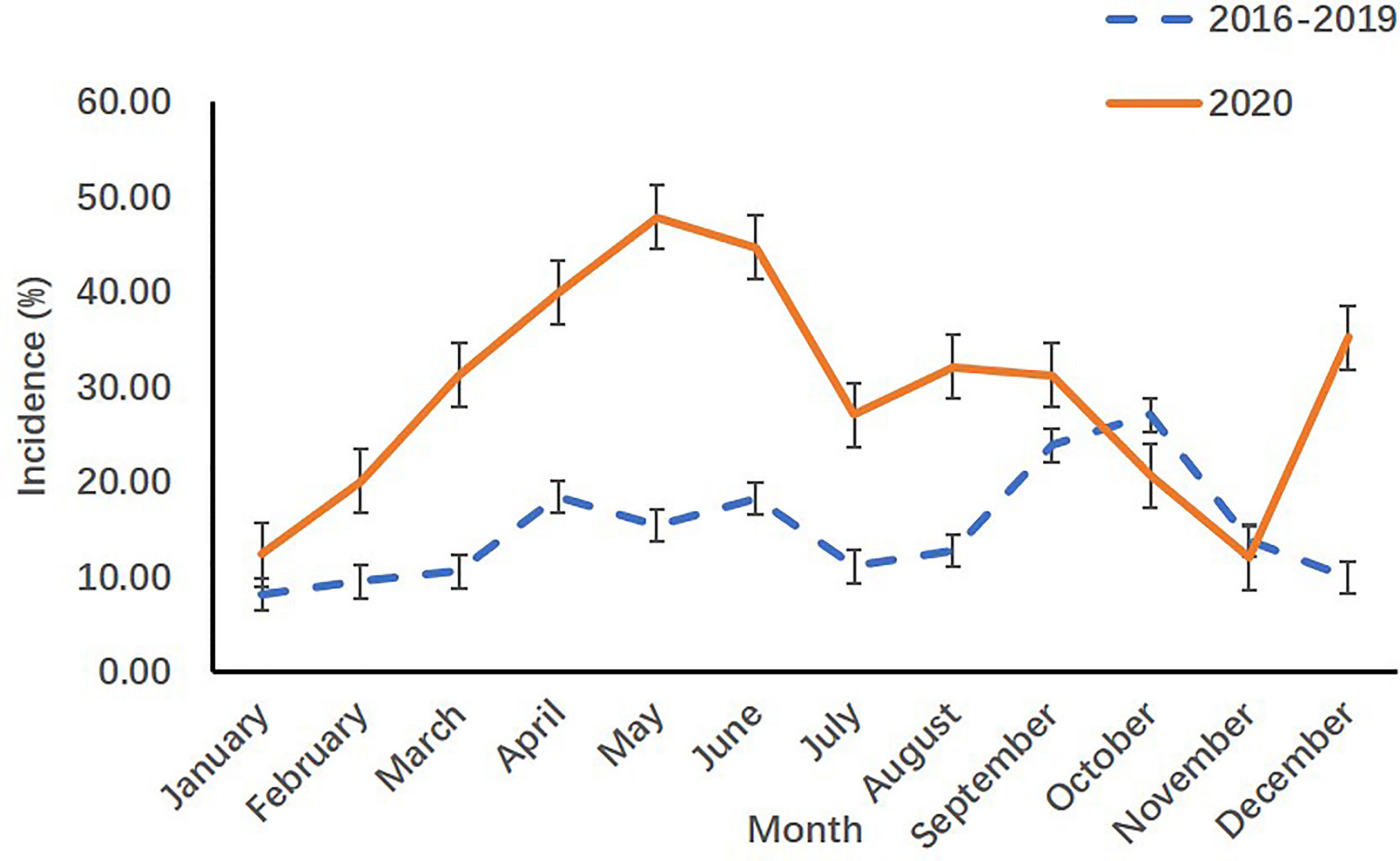
Monthly incidence rates of precocious girls before (2016-2019) and during (2020) the COVID-19 pandemic in the endocrinology ward.

When compared with the incidence in 2016-2019 years, the monthly incidence rates of precocious girls in 2020 increased from March to August and in December in the endocrinology ward of SCMC (*p*<0.05) [(**Table 2**) and (**Figure 2**)].

**Table 2:** Monthly incidence rates of precocious girls before (2016-2019) and during (2020) the COVID-19 pandemic in the endocrinology ward.

Month	Average visits of patients before (2016-2019) COVID-19 pandemic			Visits of patients during (2020) COVID-19 pandemic			*χ2*	*p*
	Precocious girls	All patients	Incidence (%)	Precocious girls	All patients	Incidence (%)		
January	3	40	8.13	8	65	12.31	0.205	0.650
February	4	45	9.44	4	20	20.00	0.722	0.396
March	6	52	10.53	19	61	31.15	6.512	**0.011**
April	9	48	18.42	49	123	39.84	6.850	**0.009**
May	8	49	15.38	66	138	47.83	15.004	**<0.001**
June	9	47	18.18	42	94	44.68	8.847	**0.003**
July	9	82	11.04	43	159	27.04	8.255	**0.004**
August	13	99	12.69	61	190	32.11	12.301	**<0.001**
September	16	68	23.81	29	93	31.18	1.142	0.285
October	16	59	27.00	20	97	20.62	0.873	0.350
November	8	56	13.78	6	50	12.00	0.097	0.755
December	5	53	9.91	25	71	35.21	10.995	**0.001**

Bold indicates p < 0.05.

**Figure 3:**
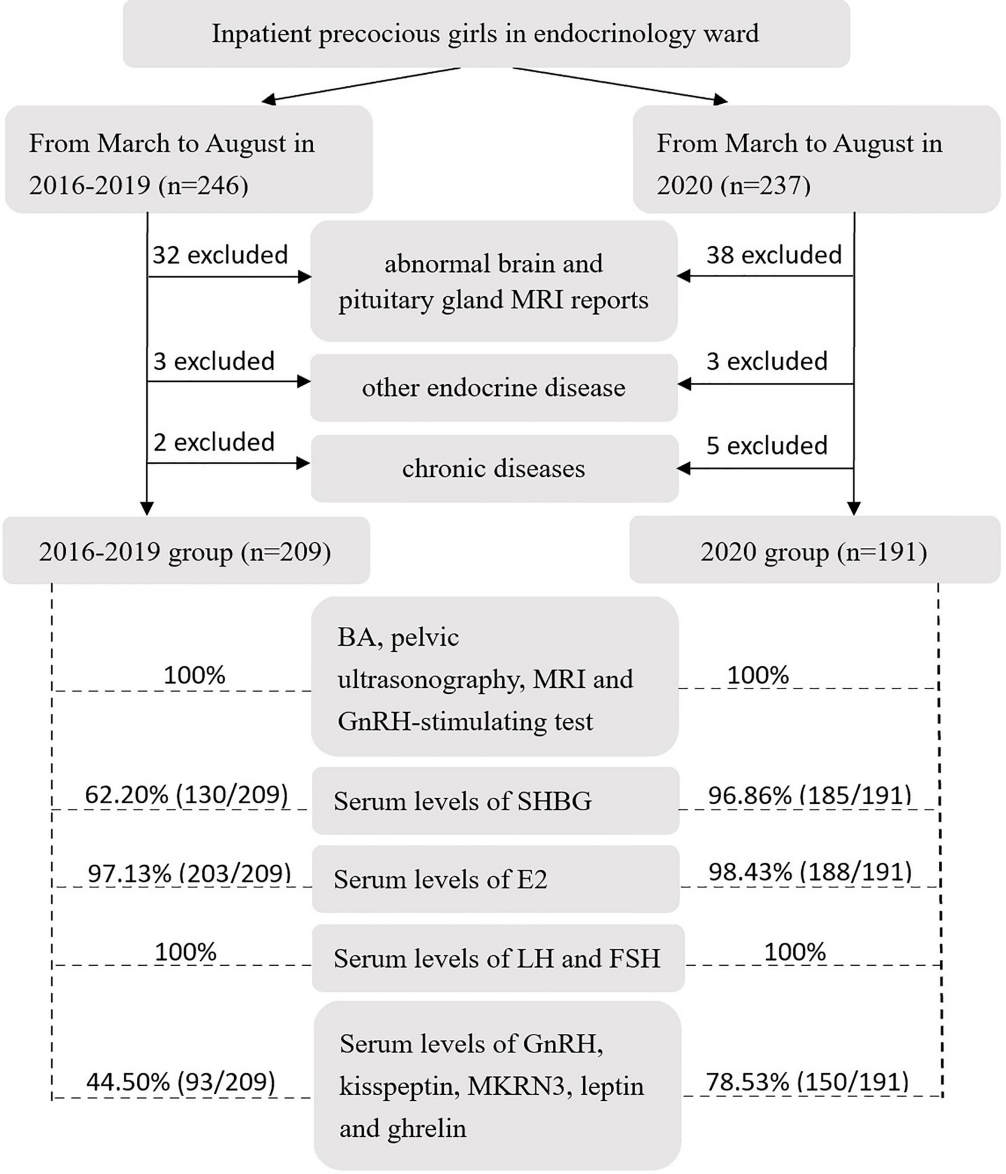
Flow diagram of criteria and examination for inpatient precocious girls in the endocrinology ward in the 2016-2019 group and 2020 group (MRI, magnetic resonance imaging; BA, bone age; GnRH, gonadotropin-releasing hormone; SHBG, sex hormone-binding globulin; E2, estradiol; LH, luteinizing hormone; FSH, follicle-stimulating hormone; MKRN3, makorin ring finger protein 3).

According to the criteria mentioned above, 209 precocious girls (age 7.92 ± 0.71 years) were included in the 2016-2019 group and 191 precocious girls (age 7.95 ± 0.77 years) were included in the 2020 group (**Figure 3**). All subjects were Han Chinese girls. The ratio of peak LH/peak FSH and the mean concentration of GnRH in the 2020 group were higher than those in the 2016-2019 group (1.23 vs 0.83, *p*<0.001 and 2.81 vs 1.99 mg/L, *p*=0.012, respectively) (**Table 3**). The mean age of mother’s menarche, the mean concentration of SHBG, basal FSH, peak FSH after the GnRH stimulation test, MKRN3 and ghrelin in the 2020 group were lower than those in 2016-2019 group (12.74 vs 13.19 years, *p*=0.024; 70.30 vs 81.64 nmol/L, *p*=0.001; 2.77 vs 3.60 IU/L, *p*<0.001; 10.66 vs 16.49 IU/L, *p*<0.001; 1.02 vs 1.93 ng/ml, *p*<0.001; 0.38 vs 0.88 ng/ml, *p*<0.001, respectively) (**Table 3**).

**Table 3:** Characteristics of inpatient precocious girls in the endocrinology ward.

Characteristics	2016-2019 group (n = 209)	2020 group (n = 191)	Statistics *Z/χ^2^*	*p*
Age (years)	7.92 ± 0.71 7.98 (6.12-8.99)	7.95 ± 0.57 7.99 (6.13-8.98)	-0.073	0.942
6<age ≤ 8	105 (50.24%)	98 (51.31%)	0.046	0.831
8<age ≤ 9	104 (49.76%)	93 (48.69%)		
BMI (kg/m^2^)	17.12 ± 2.41	17.48 ± 2.20	-1.960	0.050
Mother’s menarche age (years)	13.19 ± 1.49	12.74 ± 1.16	-2.258	**0.024**
BA-CA	1.28 ± 1.13	1.48 ± 1.13	-1.683	0.092
SHBG (nmol/L)	81.64 ± 33.28	70.30 ± 30.06	-3.254	**0.001**
Basal E2 (pg/mL)	22.31 ± 16.25	21.35 ± 13.35	-0.345	0.730
Basal LH (IU/L)	0.67 ± 0.77	0.72 ± 0.85	-0.604	0.546
Basal FSH (IU/L)	3.60 ± 1.96	2.77 ± 1.50	-4.455	**<0.001**
Peak LH (IU/L)	13.23 ± 13.79	13.30 ± 11.75	-0.576	0.564
≥5	153 (73.21%)	146 (76.44%)	0.553	0.457
<5	56 (26.79%)	45 (23.56%)		
Peak FSH (IU/L)	16.49 ± 5.69	10.66 ± 3.79	-10.842	**<0.001**
Peak LH/FSH ratio	0.83 ± 0.71	1.23 ± 0.86	-5.246	**<0.001**
GnRH (mg/L)	1.99 ± 2.03	2.81 ± 2.89	-2.514	**0.012**
Kisspeptin (ng/ml)	1.90 ± 1.38	2.13 ± 1.71	-0.345	0.730
MKRN3 (ng/ml)	1.93 ± 1.09	1.02 ± 0.84	-6.908	**<0.001**
Leptin (ng/ml)	7.14 ± 5.25	5.75 ± 2.46	-1.454	0.146
Ghrelin (ng/ml)	0.88 ± 0.43	0.38 ± 0.18	-10.719	**<0.001**

Wilcoxon rank sum tests were used for continuous variables.

χ2 tests were used for categorical variables.

Bold indicates p < 0.05.

With the peak value of LH ≥5 IU/L after the GnRH stimulation test, activation of hypothalamus-pituitary-gonadal (HPG) axis were presented in 299 precocious girls (153 in the 2016-2019 group and 146 in the 2020 group), diagnosed as central precocious puberty (15). 64.88% (194/299) of these girls showed complete records of serum concentrations of GnRH, MKRN3 and ghrelin. For these girls, no correlation was identified between serum ghrelin and serum GnRH concentrations. While, a positive correlation was found between serum ghrelin and serum MKRN3 concentrations [*β=*0.891 (*95% CI*, 0.612, 1.171); *p<*0.001] (**Table 4**).

**Table 4:** Relationships of serum ghrelin with GnRH and MKRN3 concentrations (n=194) determined by linear regression models.

Biomarkers	*β* ^a^	95% *CI*	*p*
Ghrelin	*Reference*		
GnRH (mg/L)	-0.012	-0.184-0.159	0.888
MKRN3 (ng/ml)	0.891	0.612-1.171	**<0.001**

a Models adjusted for the girls’ age, BMI and menarche age of their mothers.

Bold indicates p < 0.05.

46.67% (21/45) of precocious girls consumed much more meat than vegetables (data not shown). The ranges and percentiles of weight gain within 3 and 6 months, BMI gain within 3 and 6 months, sleep duration at night, amount of exercise, amount of outdoor activities and amount of electronic screen exposure per day of these subjects were shown in **Table 5**. The median value of weight gain in 6 months was 2 kg in these girls, the total amount of exercise per day was less than 1 hour, and the amount of electronic screen exposure per day was up to 3 hours.

**Table 5:** Lifestyle features of precocious girls during the COVID-19 pandemic.

Items	n	Range	Percentiles		
			25th	50th	75th
Weight (kg) or BMI (kg/m2) gain					
Weight gain within 3 months	24	0-4.00	0.50	1.00	2.00
BMI gain within 3 months	23	-0.68-1.96	-0.07	0.18	0.57
Weight gain within 6 months	27	0-5.00	1.50	2.00	3.63
BMI gain within 6 months	24	-1.43-2.08	-0.27	0.30	0.97
Sleep duration at night per day (h)	45	8.00-11.00	9.00	9.50	10.00
Amount of exercise per day (h)					
High intensity	44	0-1.14	0	0.07	0.29
Moderate intensity	45	0-2.14	0.14	0.29	0.43
Low intensity	42	0-2.50	0.14	0.36	1.00
Total	41	0.11-3.50	0.49	0.86	1.27
Amount of outdoor activities per day (h)	45	0-4.50	0.50	1.00	1.75
Amount of screen exposure per day (h)	45	0.50-8.00	1.59	3.00	4.75

## Discussion

In the present study, we found that the incidence rates of precocious puberty were increased and concentrations of serum MKRN3 and ghrelin were decreased during the COVID-19 pandemic among Shanghai school-aged girls. Furthermore, a positive association between the serum concentration of MKRN3 and indicated ghrelin as a potential regulatory mechanism of puberty.

The COVID-19 pandemic has spread rapidly and widely worldwide since early December (1, 2). Compared with adults, children are less susceptible to COVID-19 (3), and pediatric patients have less severe clinical manifestations (2). Studies have demonstrated that after the outbreak of COVID-19, the spectrum of disease for children changed dramatically, with an increased incidence of pubertal development problems and a decreased incidence of other infectious diseases (3). Li et al. analyzed data from Hangzhou before (from January 1, 2019 to March 31, 2019) and after (from January 1, 2020 to March 31, 2020) the breakout of COVID-19. Compared with 2019, the visit rate for problems related to precocious and accelerated puberty increased more than 3-fold during the period in 2020 (3). Stagi et al. compared medical records of precocious girls in Italy from March to July 2020 with the same period of the previous 5 years (March to July 2015-2019). An increased incidence of newly diagnosed precocious puberty in girls in 2020 and an accelerated rate of pubertal progression in precocious girls in 2020 were reported (5). In the present study, we evaluated historical data from the outpatient department and inpatient endocrinology ward in SCMC, which is one of the largest comprehensive pediatric medical centers in China, and obtained similar results. Compared with historical data in 2016-2019, the monthly incidence rates of precocious puberty in outpatient girls from February to December in 2020 (*p<*.001) and in inpatient girls from March to August in 2020 (*p<*.05) increased continually. The monthly incidence rates of precocious puberty in inpatient girls from September to November 2020 was not different from that in 2016-2019 (*p>*.05), but it increased again in December 2020 (*p=*.001), followed by a partial and transient relief of COVID-19 cases in late November in Shanghai. The decreased physical activities, increased food consumption and rapid weight gain may be associated with the increased visits of pubertal development problems during the pandemic.

SHBG is a circulating glycoprotein that transports steroid hormones in the blood (16). Most steroid hormones in the plasma are bound to proteins in the inactive bound state (17). Serum concentrations of SHBG rise significantly from birth to early childhood (18), are stable during childhood, and then decline during puberty (19). It has been hypothesized that during childhood, SHBG may restrict the actions of sex steroids in the inactive bound state and then decrease during puberty, resulting in increased levels of free sex steroids in the active unbound state (20). Increasing evidence has shown that lower concentrations of serum SHBG are associated with early puberty (19, 21). In the present study, we found that the serum concentrations of SHBG in the 2020 group were lower than those in 2016-2019 group (70.30 vs 81.64 nmol/L) (*p*<.001). The mechanism by which concentrations of serum SHBG are reduced during puberty is still unknown.

Functioning as a bridge between the hypothalamus and the gonads, FSH and LH, which are synthesized and secreted in the pituitary, play important roles in the normal function of the HPG axis (22). The secretion of FSH is regulated by a complex interplay of different factors, including negative steroid hormone feedback from the gonads, endocrine disruptor factors and stress (15). Pescovitz et al. reported that compared with girls with isolated thelarche, girls with complete sexual development showed lower serum concentrations of peak FSH after the GnRH stimulation test (23). In the present study, we found that the serum concentrations of basal FSH (2.77 vs 3.60 IU/L, *p<*.001) and peak FSH after the GnRH stimulation test (10.66 vs 16.49 IU/L, *p<*.001) were lower in the 2020 group than those in the 2016-2019 group. Although there was no difference in serum concentrations of peak LH between the two groups, the peak LH/FSH ratio, which could represent progressive puberty (23), was higher in the 2020 group than in the 2016-2019 group (1.23 VS 0.83, *p<*.001). As a cross-sectional study, we were unable to collect information about the progression of the precocious girls in our study. Data from Italy have shown an accelerated rate of pubertal progression in precocious girls in 2020 (5). This highlights the need to monitor pubertal progression for precocious girls in the 2020 group in the follow-up.

**Figure 4:**
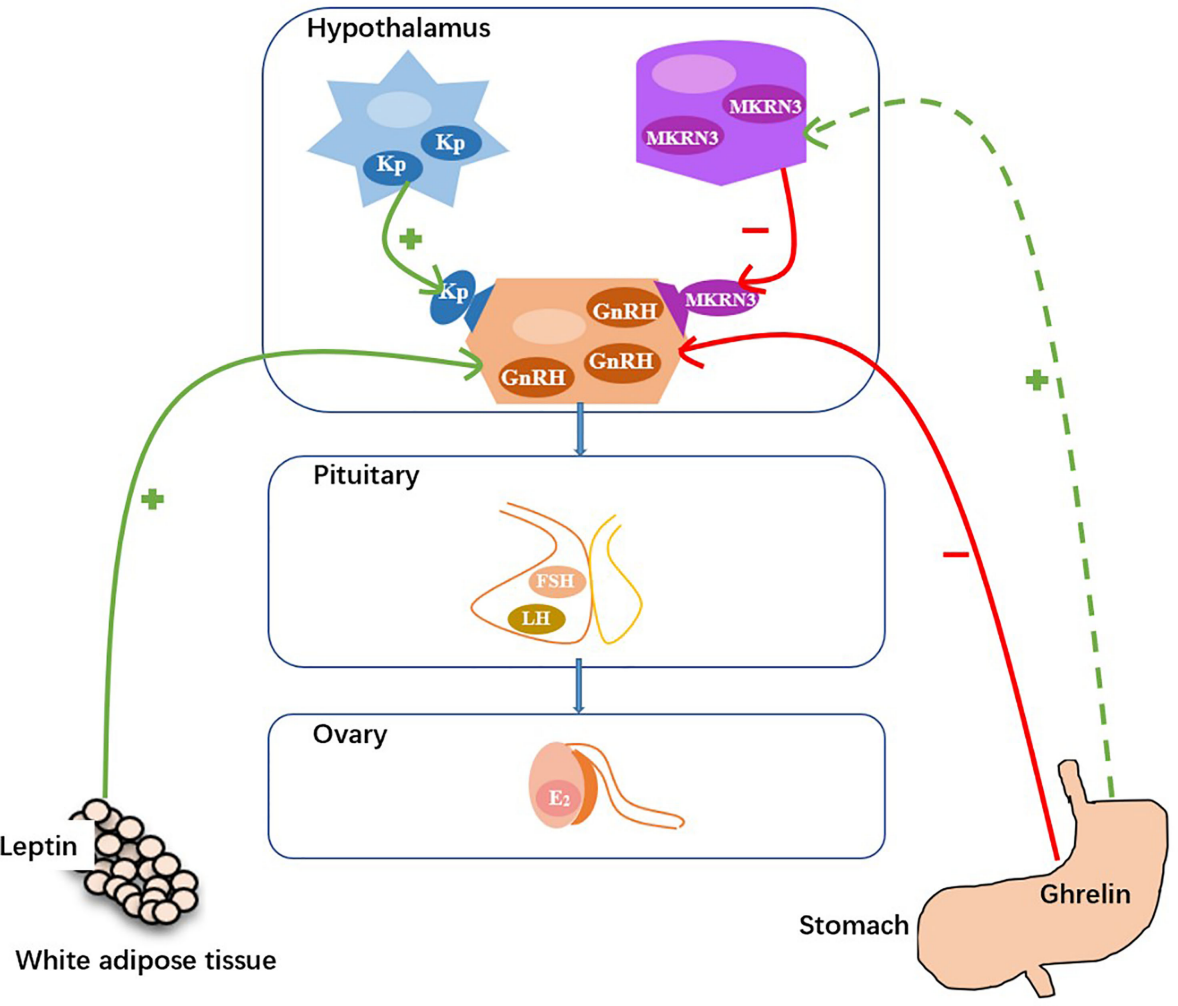
Hypothetical model for the control of GnRH secretion (Kp, kisspeptin; MKRN3, makorin ring finger protein; GnRH, gonadotropin-releasing hormone; FSH, follicle-stimulating hormone; LH, luteinizing hormone; E2, estradiol. +, stimulatory effect; −, inhibitory effect.).

Early activation of the HPG axis inducing pulsatile secretion of GnRH could give rise to early onset of puberty (24). Pulsatile secretion of GnRH is positively regulated by kisspeptin and negatively regulated by MKRN3 (**Figure 4**) (6). Compared with normal healthy girls, precocious girls showed higher concentrations of serum kisspeptin (12) and lower concentrations of serum MKRN3 (25, 26). In the present study, we found that the serum concentrations of GnRH in 2020 group were higher than those in the 2016-2019 group (2.81 vs 1.99 mg/L, *p*=.012) and the serum concentrations of MKRN3 in 2020 group were lower than those in 2016-2019 group (1.02 vs 1.93 ng/ml, *p*<.000). However, the serum concentrations of kisspeptin between the two groups were not different (2.13 vs 1.90 ng/ml, p=.730).

Under the policies of city lockdown, enforced social distancing, and school closures, the lifestyles of children changed, with less physical activities, more electronic screen exposure and increased food consumption, may lead to rapid weight gain during the pandemic (27, 28).According to questionnaires completed by 45 precocious girls and their parents in the 2020 group, the median value of weight gain in 6 months was 2 kg, and the maximum value was as high as 5 kg, which demonstrates the rapid weight gain for precocious girls in 2020 during the COVID-19 pandemic.

Rapid weight gain leads to increased concentrations of leptin and decreased concentrations of ghrelin (29). Increasing evidence has demonstrated that leptin promotes pulsatile GnRH secretion and that ghrelin suppresses pulsatile GnRH secretion, therefore they can play important roles in pubertal onset (**Figure 4**) (6, 30, 31). Kang et al. reported that serum leptin concentrations were higher in precocious girls than in normal healthy girls (30). Eshmawy et al. reported that compared with normal healthy controls, boys with constitutional delay of growth and puberty had lower serum leptin concentrations and higher serum ghrelin concentrations (31). However, few data exist on the relationship between serum ghrelin concentrations and pubertal onset in girls. We found that the serum concentrations of leptin between the two groups were not different (5.75 vs 7.14 ng/ml, *p*=.146). While, the serum concentrations of ghrelin in the 2020 group were significantly lower than those in the 2016-2020 group (0.38 vs 0.88 ng/ml, *p*<.001).

How does ghrelin contribute to the regulation of pulsatile secretion of GnRH, directly or indirectly? As far as we know, reports about that were limited. In the present study, no correlation was found between serum ghrelin and serum GnRH concentrations [*β*=-0.012 (95% *CI*, -0.184, 0.159); *p*=0.888]. While, a positive correlation was found between serum ghrelin and serum MKRN3 concentrations [*β=*0.891 (*95% CI*, 0.612, 1.171); *p<*0.001]. According to the results, we assume that the lower concentrations of ghrelin may downregulated the concentrations of MKRN3, and then upregulated the pulsatile secretion of GnRH to promote the onset of puberty (**Figure 4**). To the best of our knowledge, this is the first report of a positive correlation between serum ghrelin and serum MKRN3 concentrations in precocious girls.

## Limitations

It should be kept in mind that prolonged frozen storage of samples for the detection of GnRH, kisspeptin, MKRN3, leptin and ghrelin may lead to some extent of degradation of hormones. Although we found that precocious girls during the COVID-19 pandemic had insufficient exercise time, excessive weight gain and excessive amount of electronic screen exposure, the small number of questionnaires (23.56%) and the lack of questionnaires from precocious girls in the 2016-2019 group prevented us from analyzing the relationship between lifestyle changes and precocious puberty in girls. Moreover, as a cross-sectional study, we could not assess whether there was a causal relationship between serum ghrelin and MKRN3 concentrations. We suggest that longitudinal studies on this topic should be carried out urgently.

## Conclusions

In summary, compared with data from 2016-2019 years, the monthly incidence of precocious puberty among Shanghai school-aged girls was increased in 2020. Furthermore, a positive correlation between serum concentrations of ghrelin and MKRN3 was found, which indicated ghrelin as a potential regulatory mechanism of puberty. Longitudinal studies are needed to determine the causal relationship between ghrelin and MKRN3.

## Data Availability Statement

The original contributions presented in the study are included in the article/supplementary material. Further inquiries can be directed to the corresponding authors.

## Ethics Statement

The studies involving human participants were reviewed and approved by the institutional review board of Shanghai Children’s Medical Center, Shanghai Jiao Tong University School of Medicine. Written informed consent to participate in this study was provided by the participants’ legal guardian/next of kin. Written informed consent was obtained from the minor(s)’ legal guardian/next of kin for the publication of any potentially identifiable images or data included in this article.

## Author Contributions

YC, JC, SL and XW contributed to conception and design of the study. YT, QZ, YW, QL, XL and ZW organized the database. YC and SL performed the statistical analysis. YC and JC wrote the first draft of the manuscript. All authors contributed to manuscript revision, read, and approved the submitted version.

## Funding

This study was supported by grant 81872637, grant 81903341 and grant 82173534 from the National Natural Science Foundation of China, and grant YG2019QNA05 from the Medical and Industrial Cross Research Foundation from Shanghai Jiao Tong University.

## Conflict of Interest

The authors declare that the research was conducted in the absence of any commercial or financial relationships that could be construed as a potential conflict of interest.

## Publisher’s Note

All claims expressed in this article are solely those of the authors and do not necessarily represent those of their affiliated organizations, or those of the publisher, the editors and the reviewers. Any product that may be evaluated in this article, or claim that may be made by its manufacturer, is not guaranteed or endorsed by the publisher.
